# Out-of-pocket expenditure by Australian seniors with chronic disease: the effect of specific diseases and morbidity clusters

**DOI:** 10.1186/1471-2458-14-1008

**Published:** 2014-09-27

**Authors:** M Mofizul Islam, Laurann Yen, Jose M Valderas, Ian S McRae

**Affiliations:** Australian Primary Health Care Research Institute, Australian National University, Building 63, Cnr. Mills & Eggleston Roads, Acton ACT 2601, Canberra, Australian Capital Territory Australia; Health Services and Policy Research, University of Exeter Medical School, Exeter, England

**Keywords:** Costs, Financial stress, Long term conditions, Multimorbidity, Comorbidity, Disease cluster

## Abstract

**Background:**

Out of pocket expenditure (OOPE) on healthcare is related to the burden of illness and the number of chronic conditions a patient experiences, but the relationship of these costs to particular conditions and groups of conditions is less studied. This study examines the effect on OOPE of various morbidity groupings, and explores the factors associated with a ‘heavy financial burden of OOPE’ defined by an expenditure of over 10% of equivalised household income on healthcare.

**Methods:**

Data were collected from 4,574 senior Australians using a stratified sampling procedure by age, rurality and state of residence. Natural clusters of chronic conditions were identified using cluster analysis and clinically relevant clusters based on expert opinion. We undertook logistic regression to model the probability of incurring OOPE, and a heavy financial burden; linear regression to explore the significant factors of OOPE; and two-part models to estimate the marginal effect of factors on OOPE.

**Results:**

The mean OOPE in the previous three months was AU$353; and 14% of respondents experienced a heavy financial burden. Medication and medical service expenses were the major costs. Those who experienced cancer, high blood pressure, diabetes or depression were likely to report higher OOPE. Patients with cancer or diabetes were more likely than others to face a heavy burden of OOPE relative to income. Total number of conditions and some specific conditions predict OOPE but neither the clusters nor pairs of conditions were good predictors of OOPE.

**Conclusions:**

Total number of conditions and some specific conditions predict both OOPE and heavy financial burden but particular comorbid groupings are not useful in predicting OOPE. Low-income patients pay a higher proportion of income than the well-off as OOPE for healthcare. Interventions targeting those who are likely to face severe financial burdens due to their health could address some of these differences.

## Background

Chronic conditions are by definition long-term, and patients with such conditions often require continuing care. Responding to the care demands of people with chronic conditions is a challenge in most countries in the world [1]. Health services may impose a regressive cost burden on households [2]. Even in countries with universal healthcare coverage, patients including those with chronic conditions can still incur substantial and increasing amounts of out-of-pocket expenditure (OOPE) [3–5]. In Australia, for example, overall average OOPE increased by around 30% between 2007 and 2010-11 [6], and OOPE accounts for almost a quarter of total healthcare costs [5]. High levels of OOPE on healthcare may leave insufficient income for other necessities, and may also impede access to healthcare, affecting health status and quality of life [7]. OOPE is often felt most severely if it becomes excessive relative to income, particularly for elderly people with multiple chronic conditions who require regular and on-going engagement with the health system for the management of their health conditions [8, 9].

Apart from variation across health care settings [10–12], OOPE is likely to be influenced by a range of factors including, but not limited to, the type, total number and severity of diseases and patients’ socio-economic status [7, 12–14]. The level of this spending also varies by age and insurance coverage, among other characteristics [7]. In the literature the most prominent, among these factors, has been the total number of chronic conditions [7, 15–17] and OOPE has always been found to be directly associated to this total number. McRae et al found that not only was the total number a significant factor but that people with multiple chronic conditions tended to be from lower income groups. In addition, they found that each additional chronic condition added an estimated 46% to the likelihood of a person facing a severe financial burden due to health costs [17].

A patient’s OOPE is likely to be shaped not only by the total number of chronic conditions, but also by the type and patterns of comorbid chronic conditions, and the main focus of this study is to address this relationship. While there has been considerable study of particular comorbid combinations, including study of costs [18, 19] and OOPE [20, 21], these are targeted studies and there has been little if any work which looks at the relative impacts across a range of comorbidities. There is now a literature assessing the common natural clusters of chronic conditions that tend to co-occur [22–27]. A previous study examined the effect of some common disease clusters – with clusters defined mainly based on prevalent conditions – on OOPE [16]. However, there are many possible combinations of conditions, hence many ways of addressing multimorbid structures such as simple counts of chronic conditions, prevalent pairs or triplets, natural clusters identified using similarity measures (not with prevalence measures only), clinically relevant clusters. It is important to examine the effect of all these structures along with the effect of individual chronic conditions on OOPE to avert a fragmented and incomplete understanding of the role of comorbidity patterns. However, to our knowledge no previous study has examined the relative impact of all these combinations on the level of OOPE together in one paper. Neither has it been assessed whether any of these groupings or conditions was likely to account for a burdensome level of OOPE. The aim of this study is to examine the roles of specific chronic conditions and of comorbid structures on the level of OOPE, using a range of measures and clustering arrangements, and to explore the significant variables associated with a high burden of healthcare costs reflected by over 10% of income being expended on OOPE.

## Methods

### Study setting and health care delivery system

Australia has a publicly funded universal health care scheme known as Medicare. Residents are entitled to subsidised treatment from private medical practitioners, and for some services from nursing and allied health professionals. Australians can obtain free treatment in public hospitals, and private health insurance is available for patients preferring private services in hospital. Under the broad umbrella of Medicare there is also a Pharmaceutical Benefits Scheme (PBS), which provides subsidised prescription drugs. An estimated 80% of all prescription medicines dispensed in Australia receive subsidy via the PBS [28]. There is a concessional and a general co-payment rate, and also a safety net so that when a patient reaches the threshold their PBS patient contribution is reduced or removed [29].

### Participants

A validated questionnaire was mailed to a representative cross-section of the membership (n = 10,000) of National Seniors Australia during mid-2009. National Seniors Australia is a nation-wide organisation with 285,000 members aged over 50 years. A stratified sampling procedure by age, rurality and state of residence was applied.

Respondents were asked ‘Has a doctor ever told you that you had any of the following illnesses?’ This was followed by the list of 11 conditions and allowed for other conditions to be reported under ‘other chronic condition’. While information was collected on all conditions that lasted more than six months, the listed conditions were cancer, heart disease, high blood pressure (HBP), diabetes, arthritis, osteoporosis, asthma, bronchitis, Parkinson’s disease, depression and anxiety. As Parkinson’s disease had a very low prevalence (<2.0%), it was excluded from clustering.

The data collection method is described in detail in McRae et al. [17]. The survey and study were approved by the Australian National University Human Research Ethics Committee (no. 2009/309).

### Out-of-pocket expenditure

OOPE was defined as the total amount of own money respondents spent on both medical expenses and nonmedical expenses (e.g. transport, home care) related to care processes pertinent to healthcare. In Australia, home care includes domestic assistance, personal care and respite care and depending on individual needs may include services such as meals, transport, shopping and home maintenance [30]. Respondents were asked to report their personal OOPE during the previous three months under the main categories of health-related services, including medication, medical services, transport, medical equipment, home care and other expenses. Health insurance premiums were not included, because the focus of this study was to measure the financial burden that is directly related to out-of-pocket costs for medical care. Respondents reporting ‘do not know’ to any category (15%) were omitted from calculations of total costs. As extreme expenses such as those for housing modifications (one observation over $20,000) and very expensive hearing aids had the potential to significantly influence estimates, observations with quarterly costs of $5,000 or over were excluded when estimating total costs (removing 26 observations or ~1% of observations reporting total expenditure).

### Comorbid groups and prevalent pairs

Cluster analysis was used to establish the “natural groups” of chronic conditions – specifically, a partitional cluster analysis was undertaken using k-medoids and Yule’s Q similarity measure [27]. For modelling purposes, participants with none of the ten conditions were classified as the reference cluster.

From a disease management perspective in clinical practice [31] our clinical and content expert identified a set of clusters that, unlike partitional clusters, are not mutually exclusive. Thus a participant may belong to more than one clinically suggested cluster depending on the type of chronic conditions. Here again participants with no diseases become the reference group.

As an alternative means of addressing co-morbidity, following Schoenberg et al. [16]’s approach we created a categorical variable labeled ‘multiple morbidity’. Among those individuals with only one chronic illness HBP and arthritis were the most frequently occurring, so we categorised individuals with one condition into three sub-groups: those with HBP only, those with arthritis only, and those with only one condition but not arthritis or HBP. Participants with only two conditions were divided into five sub-groups: HBP + arthritis, HBP + diabetes, HBP + heart disease, arthritis + asthma, all other combinations of only two conditions. We constructed three sub-groups with the participants with only three conditions: HBP + arthritis + cancer, HBP + arthritis + diabetes, and all other combinations of three conditions. Although we could have used more sub-groups with two or three condition combinations, we did not go any further as the prevalence of such combinations became increasingly very small. This categorical variable ‘multiple morbidity’ contained all the above groups together with a category for those with four conditions, and a category for those with more than four conditions. The group with HBP only was selected as the reference group as it was the most common condition that tends to be associated with other conditions.

For many conditions the cost faced by a single patient with two conditions may be different from the sum of having the same two conditions separately [32], a phenomenon known as interaction, which then modifies the outcomes. The modified effect could be greater (positive interaction, synergism) or less (negative interaction, antagonism) than simple addition of the two effects [33]. To see whether there is any interaction due to having two particular conditions we used the most prevalent pairs (because there are 45 combinations of ten conditions, many of which are rare, we use conditions pairs with observed prevalence of ≥ 5%) along with the individual conditions in the regression models to examine the effect modification of these combinations.

### Other variables

A number of covariates were considered during analyses selected from analytical domains that previous studies have shown to be associated with OOPE [17]. These included socio-demographic variables such as age, sex, income, physical and mental health status reflected by SF-12 [34], region and number of ‘other chronic conditions’. The SF-12 is a widely used 12-item measure of health-related quality of life. Items are summarized into two weighted scales representing perceived impairment in role functioning associated with physical and mental health problems, with lower scores indicating greater impairment [34]. We included SF-12 measures to address participants’ physical and mental health status – as a proxy for disease severity. Income was converted to ‘household equivalent income’ using the modified Organisation for Economic Co-operation and Development (OECD) equivalence scales which apply a scale of 1 to the first adult in a household, 0.5 to the second and later adults, and 0.3 to children [35].

### Financial burden

As well as analysing OOPE, it is important to understand which groups of people face the greatest financial burdens due to their healthcare costs. For the purposes of this study, a heavy financial burden was defined as expending over 10% of equivalised household income on OOPE. Although this percentage is necessarily somewhat arbitrary, it has been used by a number of previous studies [3, 7, 17, 36].

### Approach to modelling

The distribution of the OOPE variable contains an abundance of zeros (30% of those who responded) and a highly skewed distribution of nonzero values. There are in practice two processes occurring – one which establishes a requirement to expend any OOPE on health matters, and second process which establishes the size of the OOPE conditional on it being non-zero. One approach to handling this data is to undertake two sets of regression: firstly a logistic regression exploring the probability of incurring OOPE, and secondly a linear regression of how much is spent with the subset who reported more than zero expenditure, after logarithmic transformation of that subset. These two regressions are then interpreted separately.

**Figure 1:**
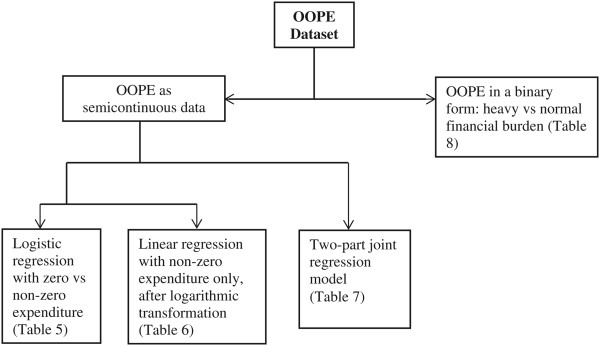
**Regression analyses for four groups of models.**

Alternatively, the two processes can be estimated jointly using an approach based on a parametric mixture distribution [37], which addresses both the abundance of zeros and the skewed distribution of non-zero values in the same model. Both of these approaches are known as two-part models, and the latter is also known as two-part joint regression model [38]. For the joint regression model we used STATA tpm command [37]. In order to show the nature of the two distinct processes as well as the overall process, in this article we used both of these approaches as shown in Figure 1. This means we report on four groups of models – (i) whether a respondent has any OOPE, (ii) the amount of OOPE for those with any OOPE, (iii) the joint modelling of the two previous models, and (iv) models based on whether the respondents faced a heavy cost burden from their conditions.

### Models estimated

For each of these four groups (i-iv mentioned above) a set of five models were estimated: model 1 estimated the association between the relevant measure of OOPE and aggregated number of chronic conditions; model 2 estimated the association between OOPE and specific chronic conditions; model 3 estimated the association between OOPE and ‘multiple morbidity’; model 4 estimated the association between OOPE and natural clusters; and model 5 estimated the association between OOPE and clinically relevant clusters.

To see the effect modification of one condition on another we also assessed the effect of prevalent pairs on both of the dependent variables. However, for the sake of parsimony we have chosen to report these results briefly in the text rather than in the tables.

Multicolinearity was assessed using variance inflation factors (VIF) and not found to be a problem. Models were compared using Akaike information criterion (AIC) and Bayesian information criterion (BIC). Data were analysed using STATA (version 12). To make the coefficients (*β*) of the models in group-ii regressions easily interpretable they have been exponentially transformed and reported as a value B. The interpretation is that a one unit (e.g. from zero to one) increase of independent variable would result in (B-1)*100 percentage change in OOPE.

The Mantel-Haenszel chi-square test for linear trend (χ^2^_trend_) was used to assess whether the proportion of participants who spent over 10% of income as OOPE showed a trend in relation to their level of household income.

## Results

### Demographic characteristics

A total of 4,574 participants completed the survey; the response rate was 45.7%, of which 43% were male and 57% female. Participants’ mean age was 69.3 years, 15 participants identified themselves as of Aboriginal or Torres Strait Islanders descent. More than three quarters (77%) were born in Australia. Over half of the participants had post school qualifications. Sixty percent were completely retired or pensioners. The participants were similar to the average Australian population of this age on most of the demographic characteristics except that the participants were better educated (certificate/diploma/university degree: 55.8% *cf* 36.6%), reported better health (excellent/very good/good: 85.8% *cf* 68.0%) and were more likely to have private insurance coverage than the average Australian in their age range (81% *cf* 57%). The sample was also similar to the average Australian population in terms of estimated prevalence of chronic conditions in the similar age group, except that the sample had higher prevalence of HBP, history of cancer diagnosis and a lower prevalence of arthritis [17].

### Prevalence of chronic diseases and comorbid conditions

Eighty-two percent of participants reported having at least one chronic condition and over 52% having at least two chronic conditions. Overall, 27% reported at least three chronic conditions, 11% have at least four and 3% have at least five conditions. HBP (43.1%), arthritis (32.2%) and asthma/hayfever (18.2%) were three most prevalent conditions (Table 1). Female participants reported a significantly higher number of conditions than male participants. Participants suffering from any chronic conditions had an average of 2.4 comorbid conditions. Table 2 shows the most frequently co-occurring pairs of conditions.

**Table 1:** **Prevalence of individual conditions, corresponding other conditions and total out-of-pocket expenditure**

Conditions	Weighted prevalence (%)*	Participants with one condition only %	Other conditions and out-of-pocket expenditure	
			Mean total number of other conditions (±SE)	Total OOPE in AUD (±SE)
High blood pressure	43.1	8.15	2.83 (0.03)	459 (53.7)
Arthritis	32.2	4.48	3.07 (0.04)	461 (31.1)
Asthma/hayfever	18.2	2.03	3.28 (0.06)	547 (56.6)
Cancer	17.9	2.88	3.06 (0.05)	702 (82.0)
Depression	15.3	1.01	3.49 (0.06)	624 (59.6)
Diabetes	12.8	1.27	3.24 (0.06)	503 (53.7)
Heart disease	12.3	1.68	3.28 (0.06)	543 (52.2)
Osteoporosis	9.3	1.11	3.38 (0.07)	465 (68.6)
Bronchitis/Emphysema	3.4	0.11	4.07 (0.12)	436 (76.3)
Stroke	3.2	0.15	3.82 (0.12)	453 (68.1)
Parkinson’s disease	0.60	0.10	3.61 (0.26)	504 (160.0)
Other	25.4	2.54	2.92 (0.05)	544 (43.4)

*Weighted to reflect the age, sex, and State structure of the Australian population.

**Table 2:** **Most frequently co-occurring pairs of conditions**

Frequently co-occurring pairs	Weighted Prevalence (%)
HBP and Arthritis	18.05
HBP and Cancer	8.77
Arthritis and asthma/hayfever	8.02
HBP and Heart disease	8.33
HBP and Asthma/hayfever	7.61
Arthritis and Cancer	6.97
HBP and Diabetes	7.98
Arthritis and Depression	6.71
HBP and Depression	6.65
Arthritis and Heart disease	6.21
Arthritis and Osteoporosis	5.62

HBP: High blood pressure.

The cluster analysis identified four natural groups depending on combinations of conditions, and we label the clusters according to the dominant conditions in each group. For instance, 46% of the participants with HBP fell in Natural Cluster 3 with the rest in Natural Cluster 1 (23%), Natural Cluster 2 (16%) and Natural Cluster 4 (15%), so for the purpose of identifying clusters participants with HBP were labelled as belonging to Natural Cluster 3. This group was also the dominant group for diabetes, and hence we describe Natural Cluster 3 as *HBP and diabetes* cluster. Clinically derived clusters are shown in the right half of Table 3.

**Table 3:** **Natural clusters from partitional cluster analysis and expert suggested clinically relevant clusters**

Natural cluster (mutually exclusive)				Clinically suggested clusters (not mutually exclusive)				
Natural Cluster 1	Natural Cluster 2	Natural Cluster 3	Natural Cluster 4	Clinical Cluster 1	Clinical Cluster 2	Clinical Cluster 3	Clinical Cluster 4	Clinical Cluster 5
Asthma	Heart	HBP	Cancer	Asthma	Arthritis	Heart	Depression	Cancer
Bronchitis	Stroke	Diabetes		Bronchitis	Osteoporosis	Stroke		
Arthritis						HBP		
Osteoporosis						Diabetes		
Depression								

### Out-of-pocket expenditure and significant factors

The mean OOPE on healthcare in the previous three months was AU$353, with a median expenditure of AU$150. Fourteen percent of the participants reported a heavy financial burden, reflected by over 10% of income being expended on OOPE. Medication and medical services expenses are the major components of these costs, but substantial costs also applied for equipment and other expenses.

Table 4 provides a summary of the significant effects in each of the models reported in Tables 5, 6, 7, and 8. The number of chronic health conditions was a significant determinant of all the different financial outcomes assessed in the modelling. For example, the likelihood of reported OOPE greater than zero increases by 44% per additional chronic disease (Table 5). Sub-group analysis also shows that among the participants who reported any expenditure the OOPE increases by 20% for an additional chronic disease (Table 6). Similar and significant associations were also observed in other two groups of regressions (Table 7 and Table 8).

**Table 4:** **Summary of significantly high out-of-pocket expenditure in all models detailed in Tables** 5, 6, 7 **and** 8

Model structure	Zero vs some OOPE: Table 5	Regression of log of outlays: Table 6	Two-part joint: Table 7	Heavy vs normal OOPE: Table 8
**Total number of conditions (Model 1)**	Highly significant	Highly significant	Highly significant	Highly significant
**Individual Conditions (Model 2)**	Cancer, heart, HBP and diabetes are significant	Cancer, HBP, diabetes and depression are significant	Cancer and asthma are significant	Cancer and diabetes are significant
**Multiple morbidity (Model 3)**	Non-specific combinations of two, three, four and more conditions are significant but not combinations of particular conditions	Non-specific combinations of three, four and more conditions are significant but not combinations of particular conditions	Non-specific combinations of conditions are significant but not combinations of particular conditions	Non-specific combinations of two, three, four and more conditions and the combination of *HBP, arthritis and diabetes* are significant
**Natural clusters (Model 4)**	All clusters significant	All clusters significant	All clusters significant	All clusters significant
**Clinical clusters (Model 5)**	4 of 5 clusters are significant	4 of 5 clusters are significant	Only cancer cluster is significant	3 of 5 clusters are significant

**Table 5:** **Logistic regression model exploring significant correlates of at least some OOPE (more than zero vs zero)**

Variable	Logistic model A1			Logistic model A2			Logistic model A3			Logistic model A4			Logistic model A5		
	OR	***p***	95% CI	OR	***p***	95% CI	OR	***p***	95% CI	OR	***p***	95% CI	OR	***p***	95% CI
**Number of chronic diseases**	1.44	<0.01	1.33-1.57												
**Specific chronic conditions**															
Cancer				1.60	<0.01	1.23-2.08									
Heart				1.54	<0.01	1.12-2.12									
HBP				1.56	<0.01	1.29-1.89									
Stroke				1.33	0.39	0.69-2.56									
Diabetes				1.43	0.04	1.02-2.00									
Asthma				1.26	0.09	0.96-1.65									
Bronchitis				0.89	0.68	0.52-1.54									
Arthritis				1.22	0.08	0.98-1.52									
Osteoporosis				1.32	0.11	0.94-1.86									
Depression				1.38	0.06	0.99-1.93									
**Multiple morbidity** ^**a**^															
No disease							0.50	<0.01	0.36-0.69						
Arthritis only							0.81	0.38	0.51-1.29						
All others with one disease							1.01	0.97	0.71-1.44						
HBP and arthritis							1.70	0.08	0.94-3.07						
HBP and diabetes							1.07	0.85	0.53-2.16						
HBP and heart disease							1.64	0.32	0.61-4.40						
Arthritis and asthma							1.00	0.99	0.45-2.26						
All others with two diseases							1.71	<0.01	1.19-2.47						
HBP, arthritis and cancer							1.67	0.36	0.55-5.01						
HBP, arthritis and diabetes							3.50	0.09	0.8-15.22						
All others with three diseases							1.82	<0.01	1.22-2.71						
All with four diseases							2.10	<0.01	1.29-3.41						
All with more than four diseases							2.32	<0.01	1.29-4.15						
**Natural clusters**															
People with none of these conditions										1.00	-	-			
Group 1 (asthma-bronchitis-arthritis-osteoporosis-depression)										2.03	<0.01	1.56-2.65			
Group 2 (HBP-Diabetes)										2.23	<0.01	1.74-2.86			
Group 3 (heart-stroke)										2.93	<0.01	2.04-4.20			
Group 4 (cancer)										2.40	<0.01	1.76-3.27			
**Clinically suggested clusters**															
Asthma and bronchitis													1.19	0.18	0.92-1.54
Arthritis and osteoporosis													1.33	<0.01	1.07-1.64
Depression													1.39	0.05	0.99-1.93
Heart, HBP, stroke and diabetes													1.87	<0.01	1.54-2.26
Cancer													1.61	<0.01	1.24-2.10
**Number of other chronic diseases**				1.74	<0.01	1.42-2.14				1.71	<0.01	1.40-2.10	1.74	<0.01	1.42-2.13
**Age groups**															
75+	1.00	-	-	1.00	-	-	1.00	-	-	1.00	-	-	1.00	-	-
50-64 years	1.03	0.82	0.79-1.36	1.04	0.76	0.79-1.38	1.09	0.56	0.82-1.43	1.05	0.74	0.80-1.38	1.05	0.74	0.79-1.38
65-74 years	1.00	0.97	0.80-1.27	1.00	0.97	0.79-1.27	1.03	0.82	0.81-1.30	1.02	0.84	0.81-1.30	1.00	0.99	0.79-1.26
**Male**	1.46	<0.01	1.21-1.76	1.40	<0.01	1.15-1.70	1.43	<0.01	1.18-1.73	1.37	<0.01	1.13-1.66	1.40	<0.01	1.15-1.69
**SF-12 physical health score**	0.95	<0.01	0.93-0.96	0.95	<0.01	0.93-0.96	0.95	<0.01	0.93-0.96	0.94	<0.01	0.92-0.95	0.95	<0.01	0.93-0.96
**SF-12 mental health score**	0.97	<0.01	0.95-0.99	0.97	<0.01	0.95-0.98	0.97	<0.01	0.95-0.98	0.96	<0.01	0.94-0.98	0.96	<0.01	0.95-0.98
**Income**															
<20 000 (ref)	1.33	0.03	1.03-1.72	1.33	0.03	1.03-1.72	1.32	0.03	1.02-1.71	1.33	0.03	1.03-1.72	1.32	0.03	1.03-1.70
20000-40000	1.64	<0.01	1.23-2.18	1.63	<0.01	1.22-2.17	1.63	<0.01	1.22-2.18	1.64	<0.01	1.23-2.19	1.63	<0.01	1.22-2.18
40000-60000	2.00	<0.01	1.39-2.86	1.97	<0.01	1.37-2.83	1.97	<0.01	1.37-2.83	1.93	<0.01	1.35-2.77	1.95	<0.01	1.36-2.80
60000-80000	2.72	<0.01	1.74-4.26	2.70	<0.01	1.72-4.24	2.79	<0.01	1.77-4.40	2.79	<0.01	1.77-4.39	2.77	<0.01	1.76-4.34
80000-100000	2.27	<0.01	1.54-3.36	2.23	<0.01	1.51-3.30	2.22	<0.01	1.50-3.29	2.23	<0.01	1.50-3.30	2.24	<0.01	1.51-3.32
**Region**															
Remote	1.00	-	-	1.00	-	-	1.00	-	-	1.00	-	-	1.00	-	-
Cities	2.06	0.02	1.13-3.74	2.07	0.02	1.14-3.77	2.23	<0.01	1.22-4.08	2.16	0.01	1.19-3.93	2.14	0.01	1.18-3.90
Regional	2.09	0.02	1.14-3.81	2.10	0.02	1.15-3.83	2.24	<0.01	1.22-4.10	2.16	0.01	1.18-3.95	2.15	0.01	1.18-3.93
	n = 3635; Pseudo R^2^ = 0.09			n = 3635; Pseudo R^2^ = 0.10			n = 3635; Pseudo R^2^ = 0.10			n = 3635; Pseudo R^2^ = 0.10			n = 3635; Pseudo R^2^ = 0.10		
	AIC = 3072; BIC = 3159			AIC = 3080; BIC = 3229			AIC = 3073; BIC = 3234			AIC = 3072; BIC = 3184			AIC = 3065; BIC = 3183		

^a^Reference group is the participants with only HBP.

**Table 6:** **Linear regression models reflecting correlates of out-of-pocket expenditure among participants who reported some expenditure**

Variable	Linear model 1			Linear model 2			Linear model 3			Linear model 4			Linear model 5		
	Β**	***P***	95% CI	Β**	***p***	95% CI	Β**	***p***	95% CI	Β**	***p***	95% CI	Β**	***p***	95% CI
**Number of chronic diseases**	1.20	<0.01	1.14-1.26												
**Specific chronic conditions**															
Cancer				1.27	<0.01	1.09-1.48									
Heart				1.19	0.06	0.99-1.42									
HBP				1.14	0.04	1.00-1.29									
Stroke				1.09	0.64	0.76-1.55									
Diabetes				1.25	0.03	1.02-1.52									
Asthma				1.18	0.06	0.99-1.40									
Bronchitis				1.33	0.09	0.95-1.87									
Arthritis				1.12	0.12	0.97-1.29									
Osteoporosis				1.19	0.13	0.95-1.48									
Depression				1.26	0.01	1.05-1.52									
**Multiple morbidity** ^**a**^															
No disease							0.92	0.55	0.70-1.21						
Arthritis only							0.98	0.90	0.66-1.44						
All others with one disease							1.21	0.16	0.93-1.59						
HBP and arthritis							1.32	0.16	0.90-1.94						
HBP and diabetes							1.21	0.47	0.72-2.03						
HBP and heart disease							0.72	0.29	0.40-1.31						
Arthritis and asthma							0.82	0.57	0.42-1.61						
All others with two diseases only							1.27	0.06	0.99-1.64						
HBP, arthritis and cancer							1.63	0.14	0.85-3.12						
HBP, arthritis and diabetes							1.79	0.09	0.91-3.50						
All others with three diseases only							1.51	<0.01	1.15-1.97						
All with four diseases only							1.92	<0.01	1.42-2.60						
All with more than four diseases							2.46	<0.01	1.77-3.41						
**Natural clusters**															
People with none of these conditions										1.00	-	-			
Group 1 (asthma-bronchitis-arthritis-osteoporosis-depression)										1.34	<0.01	1.08-1.66			
Group 2 (HBP-Diabetes)										1.22	0.05	1.01-1.49			
Group 3 (heart-stroke)										1.42	<0.01	1.11-1.81			
Group 4 (cancer)										1.52	<0.01	1.21-1.91			
**Clinically suggested clusters**															
Asthma and bronchitis													1.21	0.02	1.03-1.42
Arthritis and osteoporosis													1.12	0.12	0.97-1.29
Depression													1.27	0.01	1.06-1.53
Heart, HBP, stroke and diabetes													1.22	<0.01	1.07-1.38
Cancer													1.28	<0.01	1.09-1.49
**Number of other chronic diseases**				1.23	<0.01	1.11-1.36				1.21	<0.01	1.09-1.34	1.22	<0.01	1.11-1.35
**Age groups**															
75+	1.00	-	-	1.00	-	-	1.00	-	-	1.00	-	-	1.00	-	-
50-64 years	1.38	<0.01	1.15-1.66	1.36	<0.01	1.12-1.65	1.37	<0.01	1.13-1.65	1.37	<0.01	1.13-1.66	1.33	<0.01	1.10-1.61
65-74 years	1.15	0.09	0.98-1.35	1.14	0.11	0.97-1.35	1.14	0.11	0.97-1.34	1.15	0.09	0.97-1.36	1.13	0.15	0.96-1.33
**Male**	1.34	<0.01	1.18-1.52	1.32	<0.01	1.16-1.51	1.33	<0.01	1.17-1.51	1.30	<0.01	1.14-1.48	1.33	<0.01	1.16-1.52
**SF-12 physical health score**	0.97	<0.01	0.97-0.98	0.97	<0.01	0.96-0.98	0.97	<0.01	0.97-0.99	0.97	<0.01	0.96-0.98	0.97	<0.01	0.96-0.98
**SF-12 mental health score**	0.98	<0.01	0.97-0.99	0.98	0.01	0.97-0.99	0.98	<0.01	0.97-0.99	0.98	<0.01	0.97-0.99	0.98	0.01	0.97-1.00
**Income**															
<20 000 (ref)	1.00	-	-	1.00	-	-	1.00	-	-	1.00	-	-	1.00	-	-
20000-40000	1.09	0.40	0.89-1.32	1.10	0.38	0.90-1.33	1.10	0.39	0.89-1.33	1.10	0.34	0.90-1.34	1.09	0.40	0.89-1.33
40000-60000	1.46	<0.01	1.18-1.81	1.46	<0.01	1.18-1.81	1.48	<0.01	1.20-1.84	1.47	<0.01	1.19-1.83	1.46	<0.01	1.18-1.81
60000-80000	1.74	<0.01	1.35-2.24	1.74	<0.01	1.35-2.25	1.74	<0.01	1.34-2.25	1.72	<0.01	1.33-2.31	1.73	<0.01	1.34-2.24
80000-100000	1.79	<0.01	1.34-2.40	1.78	<0.01	1.33-2.39	1.77	<0.01	1.32-2.37	1.77	<0.01	1.31-2.37	1.77	<0.01	1.32-2.37
>100 000	2.21	<0.01	1.69-2.88	2.20	<0.01	1.68-2.88	2.19	<0.01	1.67-2.86	2.16	<0.01	1.65-2.83	2.18	<0.01	1.67-2.85
**Region**															
Remote	1.00	-	-	1.00	-	-	1.00	-	-	1.00	-	-	1.00	-	-
Cities	1.65	0.05	1.00-2.72	1.64	0.05	0.99-2.71	1.68	0.04	1.02-2.78	1.67	0.04	1.01-2.77	1.65	0.05	1.00-2.72
Regional	1.36	0.23	0.82-2.24	1.34	0.25	0.81-2.23	1.37	0.22	0.83-2.27	1.36	0.23	0.82-2.26	1.34	0.25	0.81-2.22
	n = 1595; R^2^ = 0.14			n = 1595; R^2^ = 0.14			n = 1595; R^2^ = 0.14			n = 1595; R^2^ = 0.13			n = 1595; R^2^ = 0.14		
	AIC = 5247; BIC = 5322			AIC = 5263; BIC = 5392			AIC = 5260; BIC = 5400			AIC = 5279; BIC = 5376			AIC = 5262; BIC = 5365		

^a^Reference group is the participants with only HBP; ** Exponentiated values of the coefficients estimated in the linear model of the logarithm of the OOPE.

**Table 7:** **Two part joint regression models reflecting marginal effects of associated variables on out of pocket expenditure**

Variable	TPM model 1			TPM model 2			TPM model 3			TPM model 4			TPM model 5		
	dy/dx	***p***	95% CI	dy/dx	***p***	95% CI	dy/dx	***p***	95% CI	dy/dx	***p***	95% CI	dy/dx	***p***	95% CI
**Number of chronic diseases**	89.1	**	53.4, 124.9												
**Specific chronic conditions**															
Cancer				257.7	**	151.2, 364.1									
Heart				70.3		-44.1, 184.7									
HBP				28.5		-51.8, 108.7									
Stroke				0.5		-229.3, 230.3									
Diabetes				84.4		-41.7, 210.6									
Asthma				112.1	*	0.7, 223.5									
Bronchitis				4.6		-213, 222.2									
Arthritis				0.6		-90.8, 92.1									
Osteoporosis				43.2		-97.8, 184.2									
Depression				75.5		-47.2, 198.1									
**Multiple morbidity** ^**a**^															
No disease							-95.2		-206.6, 16.3						
Arthritis only							-40.6		-203.9, 122.8						
All others with one disease							198.6	*	38.7, 358.5						
HBP and arthritis							41.3		-148.5, 231.0						
HBP and diabetes							9.7		-232.3, 251.7						
HBP and heart disease							-100.1		-294.7, 94.4						
Arthritis and asthma							-154.9		-328.7, 18.9						
All others with two diseases only							169.6	*	31.6, 307.6						
HBP, arthritis and cancer							63.0		-285.3, 411.3						
HBP, arthritis and diabetes							233.3		-273.8, 740.4						
All others with three diseases only							151.3	*	6.4, 296.1						
All with four diseases only							251.5	*	55.1, 447.9						
All with more than four diseases							552.0	**	241.3, 862.7						
**Natural clusters**															
People with none of these conditions										1.00		-			
Group 1 (asthma-bronchitis-arthritis-osteoporosis-depression)										139.3	*	33.0, 245.5			
Group 2 (HBP-Diabetes)										123.4	*	27.2, 219.5			
Group 3 (heart-stroke)										187.9	**	52.8, 322.9			
Group 4 (cancer)										377.5	**	215.9, 539.0			
**Clinically suggested clusters**															
Asthma and bronchitis													97.6		-7.1, 202.3
Arthritis and osteoporosis													-3.1		-92.0, 85.9
Depression													77.6		-44.2, 199.5
Heart, HBP, stroke and diabetes													63.3		-17.5, 144.1
Cancer													255.0	**	150.2, 359.8
**Number of other chronic disease**				146.9	**	83.1, 210.6				147.9	**	84.2, 211.6	146.8	**	83.5, 210.1
**Age groups**															
75+	1.00	-	-	1.00		-	1.00	-	-	1.00		-	1.00		-
50-64 years	115.9		-7.18, 238.9	78.6		-37.8, 195.0	112.4	*	0.1, 224.8	82.5		-30.5, 195.6	69.6		-43.5, 182.7
65-74 years	92.9		-8.11, 193.9	72.9		-22.8, 168.6	91.3		-2.0, 184.6	71.1		-23.6, 165.7	66.4		-28.5, 161.3
**Male**	141.1	**	46.2, 236.1	128.9	**	40.7, 217.0	145.0	**	58.5, 231.4	123.1	**	37.2, 209.0	127.5	**	41.0, 214.1
**SF-12 physical health score**	-11.9	**	-19.1, -4.8	-12.0	**	-18.5, -5.5	-12.4	**	-19.0, -5.9	-12.6	**	-19.0, -6.3	-12.4	**	-18.8, -5.9
**SF-12 mental health score**	-12.2	**	-20.4, -4.1	-11.8	**	-19.6, -4.1	-11.9	**	-19.3, -4.6	-14.3	**	-21.6, -7.0	-12.2	**	-19.9, -4.6
**Income**															
<20 000 (ref)	1.00	-	-	1.00	-	-	1.00	-	-	1.00			1.00	-	-
20000-40000	-22.7		-130.8, 85.4	1.5		-92.7, 95.7	-6.1		-102.5, 90.3	5.7		-86.6, 98.1	1.6		-91.7, 94.9
40000-60000	126.7		-11.5, 264.9	150.6	*	27.6, 273.7	146.0	*	21.4, 270.6	154.3	*	34.6, 273.9	152.4	*	31.2, 273.6
60000-80000	239.8	*	41.7, 437.9	265.4	**	86.8, 444.0	267.4	*	82.7, 452.1	255.6	**	82.2, 429.0	268.9	**	91.0, 446.8
80000-100000	272.3	*	23.0, 521.6	256.7	*	44.1, 469.2	290.4	*	62.9, 517.8	307.4	**	81.5, 533.3	264.8	*	53.1, 476.5
>100 000	397.4	**	140.7, 654.1	397.0	**	174.1, 619.9	363.2	**	145.7, 580.7	398.4	**	177.7, 619.1	401.7	**	178.8, 624.6
**Region**															
Remote	1.00	-	-	1.00		-	1.00			1.00			1.00		-
Cities	213.7	*	3.5, 423.9	228.3	*	47.1, 409.4	246.5	*	71.0, 422.0	225.2	*	46.0, 404.3	232.1	*	55.5, 408.7
Regional	164.7		-45.7, 375.2	178.2		-3.8, 360.2	179.7	*	5.1, 354.3	183.3	*	2.8, 363.8	182.0	*	4.7, 359.3
	n = 2226			n = 2226			n = 2226			n = 2226			n = 2226		
	AIC = 25827; BIC = 25987			AIC = 25782; BIC = 26056			AIC = 25777; BIC = 26074			AIC = 25784; BIC = 25989			AIC = 25761; BIC = 25978		

** < 0.01, * < 0.05; ^a^Reference group is the participants with only HBP.

**Table 8:** **Logistic regression model exploring significant correlates for respondents spending over 10% of income on health**

Variable	Logistic model B1			Logistic model B2			Logistic model B3			Logistic model B4			Logistic model B5		
	OR	***p***	95% CI	OR	***p***	95% CI	OR	***p***	95% CI	OR	***p***	95% CI	OR	***p***	95% CI
**Number of chronic diseases**	**1.36**	**<0.01**	**1.24-1.49**												
**Specific chronic conditions**															
Cancer				1.38	0.04	1.02-1.87									
Heart				1.27	0.17	0.91-1.79									
HBP				1.09	0.50	0.84-1.42									
Stroke				0.95	0.88	0.47-1.91									
Diabetes				1.79	<0.01	1.25-2.57									
Asthma				1.32	0.10	0.95-1.85									
Bronchitis				1.59	0.11	0.90-2.81									
Arthritis				1.19	0.23	0.90-1.58									
Osteoporosis				1.13	0.58	0.73-1.77									
Depression				1.33	0.12	0.93-1.89									
**Multiple morbidity** ^**a**^															
No disease							0.81	0.54	0.41-1.60						
Arthritis only							1.40	0.43	0.60-3.28						
All others with one disease							1.51	0.21	0.79-2.87						
HBP and arthritis							1.51	0.37	0.62-3.67						
HBP and diabetes							2.31	0.10	0.86-6.17						
HBP and heart disease							0.41	0.40	0.05-3.27						
Arthritis and asthma							b	b	b						
All others with two diseases							1.87	0.04	1.02-3.42						
HBP, arthritis and cancer							1.11	0.90	0.23-5.33						
HBP, arthritis and diabetes							4.98	0.01	1.51-16.41						
All others with three diseases							1.87	0.05	1.00-3.51						
All with four diseases							2.71	<0.01	1.40-5.23						
All with more than four diseases							5.08	<0.01	2.61-9.90						
**Natural clusters**															
People with none of these conditions										1.00	-	-			
Group 1 (asthma-bronchitis-arthritis-osteoporosis-depression)										1.59	0.05	1.01-2.53			
Group 2 (HBP-Diabetes)										1.66	0.02	1.07-2.58			
Group 3 (heart-stroke)										1.74	0.03	1.05-2.88			
Group 4 (cancer)										1.82	0.02	1.12-2.94			
**Clinically suggested clusters**															
Asthma and bronchitis													1.41	0.03	1.03-1.92
Arthritis and osteoporosis													1.16	0.29	0.88-1.54
Depression													1.33	0.12	0.93-1.89
Heart, HBP, stroke and diabetes													1.33	0.04	1.02-1.73
Cancer													1.37	0.04	1.02-1.86
**Number of other chronic diseases**				1.65	<0.01	1.38-1.98				1.60	<0.01	1.34-1.92	1.73	0.00	1.33-2.26
**Age groups**															
75+	1.00	-	-	1.00	-	-	1.00	-	-	1.00	-	-	1.00	-	-
50-64 years	1.07	0.71	0.74-1.54	0.97	0.86	0.66-1.42	1.04	0.83	0.72-1.51	0.98	0.90	0.67-1.41	0.96	0.84	0.66-1.40
65-74 years	1.29	0.11	0.94-1.78	1.23	0.22	0.89-1.70	1.31	0.10	0.95-1.80	1.23	0.20	0.89-1.69	1.22	0.23	0.88-1.67
**Male**	1.77	<0.01	1.36-2.29	1.70	<0.01	1.29-2.23	1.71	<0.01	1.32-2.22	1.65	<0.01	1.27-2.15	1.73	<0.01	1.33-2.26
**SF-12 physical health score**	0.97	<0.01	0.95-0.99	0.97	<0.01	0.95-0.99	0.97	<0.01	0.95-0.99	0.96	<0.01	0.94-0.98	0.97	<0.01	0.95-0.98
**SF-12 mental health score**	0.97	0.01	0.95-0.99	0.97	0.01	0.95-0.99	0.97	0.01	0.95-0.99	0.96	<0.01	0.94-0.99	0.97	0.01	0.95-0.99
**Region**															
Remote	1.00	-	-	1.00	-	-	1.00	-	-	1.00	-	-	1.00	-	-
Cities	2.87	0.09	0.84-9.82	2.92	0.09	0.84-10.19	3.00	0.08	0.87-10.39	2.80	0.10	0.82-9.49	2.79	0.10	0.82-9.51
Regional	2.68	0.12	0.78-9.19	2.75	0.11	0.78-9.62	2.75	0.11	0.79-9.56	2.58	0.13	0.76-8.81	2.58	0.13	0.75-8.85
	n = 2227; Pseudo R^2^ = 0.08			n = 2227; Pseudo R^2^ = 0.09			n = 2203; Pseudo R^2^ = 0.08			n = 2227; Pseudo R^2^ = 0.07			n = 2227; Pseudo R^2^ = 0.08		
	AIC = 1657; BIC = 1709			AIC = 1665; BIC = 1773			AIC = 1662; BIC = 1776			AIC = 1674; BIC = 1748			AIC = 1665; BIC = 1745		

^a^Reference group is the participants with only HBP; b: Not calculated due to zero cell value.

Examination of individual conditions reveals that different subsets of diseases from the set comprising cancer, HBP, diabetes, depression, heart and asthma were significant in one or more approaches. Cancer is the only condition common in all approaches.

The results of Model 3 addressing the combined multi-morbidity structure show predominantly that the non-specific combinations of two, three, four and more diseases are significantly different to the omitted category of HBP only. The only exception was in the model which examines the factors correlated with the burden of OOPE, where the specific combination with HBP, arthritis and diabetes was also significant (Table 8).

The “natural clusters” were clearly well defined as all of them were significantly different to the “having no chronic conditions” reference group under each approach to modelling, but the groupings were never significantly different one from another. The clinically defined conditions again were generally associated with having significantly greater OOPE than having no chronic conditions, while not significantly different one from another. The two exceptions were that for the model identifying those who did and did not have any OOPE the cardiovascular cluster was the most likely to face costs, and under the two-part joint modelling the cancer group was the only cluster significantly different to the no condition group, and was different to most other clusters.

Other factors that were significantly associated with the OOPE and/or OOPE burden were predominantly gender, physical and mental health status reflected in the SF-12 score and income, with rurality being significant in three groups of regressions (Tables 5, 6 and 7) and age in two (Table 6 and Table 7). OOPE among those who reported some expenditure was likely to be higher for participants who were relatively young, male, had poor physical and mental health as reflected in the SF-12, had high income and lived in urban areas.

The probability of heavy financial burden declines significantly with increasing levels of income (χ^2^_trend_ = 4.87, *p* = 0.03). None of the prevalent pairs tested were significant in the linear regressions, suggesting the absence of effect modification on OOPE by one disease on another, at least in the common combinations.

For all approaches, the AIC/BIC measures are similar across the 5 equations, but are lowest for the first equation suggesting that the equation based only on number of conditions provides as good a measure of fit as any of the equations which include information on specific conditions after accommodating parsimony.

## Discussion

The examination of clusters and dominant pairs did not give any clear discrimination between groups of conditions, except for supporting the conclusion that some individual conditions do stand out. The ‘multiple morbidity’ variable, which we developed following the approach of Schoenberg et al. [16], showed that the non-specific combinations of two, three, four and more conditions offer more strongly predictive information than the combination of specific conditions. Together these findings suggest that at least in our dataset the total number of chronic diseases and individual chronic diseases offer better and more useful information about OOPE and heavy financial burden than the clusters, dominant groups or dominant pairs. Clearly, among those who reported some expenditure OOPE increases with increasing number of chronic conditions, and it is significantly higher among those with cancer, depression, diabetes or HPB than those who did not have of these diseases.

Total number of diseases is clearly a significant determinant of OOPE and of the heavy financial burden. While this is to be expected as more conditions means more doctor visits and probably to several doctors, more tests and more medications, the fact that this dominates over any particular disease combinations is important. While there are not necessarily easy solutions to the pressures of multi-morbidity, health professionals dealing with multi-morbid patients need to be aware of these potential financial pressures in proposing treatments, and policy makers need to be aware of the growing pressures on both personal and government budgets.

Among the individual conditions, cancer in particular led to significant OOPE under all constructs followed by diabetes, which was found significant in three of the four groups of modelling. Other conditions became significant in different models. Some conditions are close to significant using some methods and become significant using other methods. In particular it is of interest that the two-part joint modelling suggests asthma is a marginally (*p* = 0.049) significant determinant of outlays, while this does not arise for any of the other approaches. This may have arisen as the combined effect of the probability of spending any amount (*p* = 0.09; Table 5) and the relative amount of spending among those who reported any spending (*p* = 0.06; Table 6), and the relatively high OOPE for patients with asthma presented in Table 1 ($547).

As noted above, cancer stands out as the condition which accounts for significantly higher OOPE than the other chronic conditions. Comparison of clusters also indicates that the cancer dominated cluster is the most strongly associated with OOPE, and has the largest impact on OOPE burden, although the differences are not significant. This observation has substantial implications for health care financing and management, as many categories of cancers – once identified as lethal diseases – now increasingly became manageable chronic diseases due to earlier diagnosis and improved treatments. Sub-analysis of our data shows that cancer patients are more likely to report significantly more OOPE in medical consultation and tests than other categories of OOPE. This is consistent to the literature, as some diagnostic tests and essential items such as MRI, PET (a common cancer scan), bone density scans, wigs to cover the balding head, special inner garments and some cancer drugs are not covered by the Medicare [39]. Also the gap payments for some tests, drugs and services are another source of OOPE. A previous study with cancer patients in rural Queensland also reported substantial OOPE on medical consultations and tests (14%) [10]. Together these findings warrant further research as to how this OOPE can be reduced among cancer patients.

Diabetes is also significantly associated with the overall OOPE and high financial burden of healthcare costs, and is a component of one of the clusters which is significant in assessing financial burden. Patients with diabetes reported significantly more OOPE than others in two major categories: *medications* and *equipment*. This is understandable given that a person with diabetes may incur OOPE on a range of supplies and medications, such as syringes, lancets, glucose testing meters, test strips, insulin pumps, insulin and/or other medications. Although all these supplies and medications are highly subsidised and some are subject to a maximum annual payment cap, the gap amount for such items on a regular basis may be substantial relative to income. The ‘front loading’ structure in Australia, where co-payments are made until the cap is reached, after which prescriptions are further subsidised (for people on higher incomes) or free (for people on lower incomes), may lead to some patients deferring or avoiding having prescriptions filled to avoid costs. Further, it must be noted that non-prescription medicines are not subsidised, and for many people these will represent a cost burden in its own right.

One of the strengths of our study is its range of approaches of disease groupings. Their associations with the outcome variables, to some extent, depend on the way they were grouped. For instance, natural clusters were identified based on distance measure (Yule’s Q); the specific groupings in ‘multiple morbidity’ and prevalent pairs were based on simple probability of association and the clinical clusters were based on ‘concordant comorbidity’. Moreover, natural clusters were mutually exclusive but the clinical clusters were not. While the groupings of conditions in clusters or in the most prevalent groups only assist in a minor way in identifying those facing the greatest financial burdens, these groupings may be of value for other purposes. The knowledge of the structure of combinations of chronic conditions may, for example, impact on matters such as time use which we are exploring in further research. Also future research should focus the evidence base on which to formalise groupings which can be more widely used to assist in our understanding of the implications of different comorbidities.

Levels of OOPE in Australia are high by high-income country standards [40, 41]. International comparison of adults with chronic conditions from eleven OECD countries in 2011 found that around one third of Australians avoided some component of care due to cost issues in the past year. This was a higher proportion than citizens of any other member country, except the USA [40]. A previous study showed that people with multimorbidity are likely to have relatively low income, and demonstrated the impact of chronic conditions on the proportion of equivalised income expended on health is more extreme than the impact on the level of expenditure. Thus cost burden falls most heavily on those with multiple chronic conditions [42] and those least able to bear it [43] – a group who are vulnerable both in terms of health and income. It is clear that senior Australians, even with the protection of Medicare, may face a burden of substantial OOPE.

One of the main findings of our study is that male patients with multiple chronic conditions, particularly cancer or diabetes, who had poor physical and mental health as reflected in the SF-12, had relatively high income, and lived in city areas are more likely to report higher OOPE. While our data are limited and do not explain the reasons for this observation, the possible explanation of this is likely to be multifaceted. Firstly, it is understandable, as our results suggest, that people with multiple chronic conditions and more severe diseases particularly cancer or diabetes are likely to incur more OOPE. Secondly, participants with relatively high income level have a tendency to enjoy better care and to be ready to pay more. For instance, they are likely to prefer to see specialist doctors in private practice, with relatively high consulting fees, than to ‘wait their turn’ in the public system. Although there is a provision of rebate through the Medicare scheme, the gap amount is borne by the patient and/or the private health insurance. Thirdly, high income patients do not enjoy all the concessions that the low-income patients do. Fourthly, available health care facilities in the city areas, unlike remote areas where there are limited opportunities, may influence the OOPE. Although for many services costs are higher in rural areas, overall expenditure is determined by the volume of services which is lower in rural areas. Finally, as males on average die significantly earlier than females in Australia and all patients face higher costs in their last years of life, this may impact on the higher costs observed for males than females.

Although people with relatively high income pay more OOPE than people with relatively low income, low income patients are more likely to face a heavy financial burden related to OOPE. Findings therefore suggest that despite a healthcare system that provides universal coverage and a well-established and extensive system of social security, some individuals with chronic illness face substantial cost burden, which falls most heavily on those least able to bear it. As household economic burden is skewed toward specific patient groups; effective remedies could include focused interventions such as income support and subsidies [11]. Our observation, for example that, male patients with multiple chronic conditions particularly cancer or diabetes and who had poor physical and mental health are likely to face a heavy burden of OOPE (Table 8) could point to one sub-group for targeted intervention. While such fine targeting may not be practicable, further research on the effects of burdensome costs would be helpful in refining supportive policies. Previous studies in Australia have shown that people defer or avoid filling prescriptions because of cost [44], although it is unknown whether they avoid both prescription and non-prescription drugs, and what choices they make in this regard.

### Limitations

We used a cross-sectional design to recruit participants and thus are unable to assess change in financial circumstances over time, or to understand the degree to which financial burdens trend upwards or downwards with changing health conditions. The expenditure data is based on recall, and perhaps more importantly the relatively short recall period needed to minimise recall error which will lead to high variability for conditions that have less frequent but expensive events. The response rate of 45.7% is highly acceptable for a mail survey in the Australian context, and leads to a reasonably large sample of 4,570 respondents. However, in combination with the structure of the National Seniors Australia membership this response rate leads to a risk that the sample may not perfectly represent the wider population of older Australians. Our sample is better educated than the wider Australian populations of same age, although their health conditions broadly reflect those of the wider population. Testing suggests that any biases from these effects are minimal, particularly as we are exploring relationships rather than estimated prevalences or dollar amounts. Our sample had only 15 participants who identified themselves as indigenous which is significantly less than the proportion of the population in this age range. We believe this is principally due to the relatively low proportion of Indigenous Australians who are members of the National Seniors Australia organisation, and hence the relatively small numbers in our sample frame. The OOPE and its corresponding effects will be different in a health care system in another country that has a different costs base. For example, in Australia a safety net based on income and total annual expenditure applies. Thus our findings may not be generalizable to other settings, and all aspects of OOPE may not be applicable for other chronic conditions. But the models we have developed here could be used more generally to identify where policy interventions can be most protective.

## Conclusion

The ability to identify patients who incur excessive OOPE is useful for appropriate policy formulation. OOPE is common among senior Australians with chronic conditions and this expenditure increases with number of chronic conditions. The poor pay a higher proportion of their income as OOPE than the well-off. Among the individual conditions, the OOPE is significantly higher for patients with cancer than patients with any other condition. HBP, diabetes, depression are also important conditions in terms of relatively high OOPE. Patients with cancer or diabetes are more likely to spend over 10% of their household income on health related purchases. With the set of conditions tested in this study, disease clusters do not provide a means to estimate OOPE. As the burden of healthcare costs on individuals is likely to continue to increase, policies are needed targeting patients with the most conditions, with cancers, and those on low incomes as they are most likely to face severe financial burdens due to their healthcare.

## Abbreviations

AIC: Akaike information criterion
AU$: Australian dollar
BIC: Bayesian information criterion
HBP: High blood pressure
OECD: Organisation for Economic Co-operation and Development
OOPE: Out-of-pocket expenditure
PBS: Pharmaceutical Benefit Scheme
SF-12: Short form-12 health survey
STATA: Statistical software created by StataCorp
USA: United States of America
VIF: Variance inflation factors.

## References

[1] Bhojani U, Thriveni B, Devadasan R, Munegowda C, Devadasan N, Kolsteren P, Criel B (2012). Out-of-pocket healthcare payments on chronic conditions impoverish urban poor in Bangalore. India. BMC Public Health.

[2] Thuan NT, Lofgren C, Chuc NT, Janlert U, Lindholm L (2006). Household out-of-pocket payments for illness: evidence from Vietnam. BMC Public Health.

[3] Essue B, Kelly P, Roberts M, Leeder S, Jan S (2011). We can’t afford my chronic illness! The out-of-pocket burden associated with managing chronic obstructive pulmonary disease in western Sydney, Australia. Journal of health services research & policy.

[4] Organisation for Economic Co-operation and Development: **Health at a Glance 2013: OECD Indicators.** http://www.oecd.org/els/health-systems/Health-at-a-Glance-2013.pdf

[5] Yusuf F, Leeder SR (2013). Can’t escape it: the out-of-pocket cost of health care in Australia. Med J Aust.

[6] Consumers Health Forum of Australia: **Australian healthcare — out of pocket and out of date?** *Health Voices* 2013, (12)**:**1–36.

[7] Hwang W, Weller W, Ireys H, Anderson G (2001). Out-of-pocket medical spending for care of chronic conditions. Health affairs.

[8] Lehnert T, Heider D, Leicht H, Heinrich S, Corrieri S, Luppa M, Riedel-Heller S, Konig HH (2011). Review: health care utilization and costs of elderly persons with multiple chronic conditions. Medical care research and review: MCRR.

[9] Piette JD, Heisler M, Wagner TH (2004). Cost-related medication underuse: do patients with chronic illnesses tell their doctors?. Arch Intern Med.

[10] Gordon L, Ferguson F, Chambers S, Dunn J (2009). Fuel, beds, meals and meds: out-of-pocket expenses for patients with cancer in rural Queensland. The Cancer Forum.

[11] Jan S, Essue BM, Leeder SR (2012). Falling through the cracks: the hidden economic burden of chronic illness and disability on Australian households. Med J Aust.

[12] You X, Kobayashi Y (2011). Determinants of out-of-pocket health expenditure in China: analysis using China Health and Nutrition Survey data. Applied health economics and health policy.

[13] Corrieri S, Heider D, Matschinger H, Lehnert T, Raum E, Konig HH (2010). Income-, education- and gender-related inequalities in out-of-pocket health-care payments for 65+ patients - a systematic review. International journal for equity in health.

[14] Bernard DS, Farr SL, Fang Z (2011). National estimates of out-of-pocket health care expenditure burdens among nonelderly adults with cancer: 2001 to 2008. Journal of clinical oncology: official journal of the American Society of Clinical Oncology.

[15] Paez KA, Lan Zhao L, Hwang W (2009). Rising Out-Of-Pocket Spending For Chronic Conditions: A Ten-Year Trend. Health affairs.

[16] Schoenberg NE, Kim H, Edwards W, Fleming ST (2007). Burden of common multiple-morbidity constellations on out-of-pocket medical expenditures among older adults. The Gerontologist.

[17] McRae I, Yen L, Jeon YH, Herath PM, Essue B (2012). Multimorbidity is associated with higher out-of-pocket spending: a study of older Australians with multiple chronic conditions.

[18] Egede LE, Zheng D, Simpson K (2002). Comorbid depression is associated with increased health care use and expenditures in individuals with diabetes. Diabetes Care.

[19] Richardson LP, Russo JE, Lozano P, McCauley E, Katon W (2008). The effect of comorbid anxiety and depressive disorders on health care utilization and costs among adolescents with asthma. General hospital psychiatry.

[20] Okumura Y, Ito H (2013). Out-of-pocket expenditure burdens in patients with cardiovascular conditions and psychological distress: a nationwide cross-sectional study. General hospital psychiatry.

[21] Davidoff AJ, Erten M, Shaffer T, Shoemaker JS, Zuckerman IH, Pandya N, Tai MH, Ke X, Stuart B (2013). Out-of-pocket health care expenditure burden for Medicare beneficiaries with cancer. Cancer.

[22] Cornell JE, Pugh JA, Williams JW, Kazis L, Lee AF, Parchman ML, Zeber J, Montgomery KA, Nokl PH (2007). Multimorbidity clusters: clustering binary data from multimorbidity clusters:clustering binary data from a large administrative medical database. Applied Multivariate Research.

[23] Garcia-Olmos L, Salvador CH, Alberquilla A, Lora D, Carmona M, Garcia-Sagredo P, Pascual M, Munoz A, Monteagudo JL, Garcia-Lopez F (2012). Comorbidity patterns in patients with chronic diseases in general practice. PLoS One.

[24] Steinman MA, Lee SJ, John Boscardin W, Miao Y, Fung KZ, Moore KL, Schwartz JB (2012). Patterns of multimorbidity in elderly veterans. J Am Geriatr Soc.

[25] Prados-Torres A, Poblador-Plou B, Calderon-Larranaga A, Gimeno-Feliu LA, Gonzalez-Rubio F, Poncel-Falco A, Sicras-Mainar A, Alcala-Nalvaiz JT (2012). Multimorbidity patterns in primary care: interactions among chronic diseases using factor analysis. PLoS One.

[26] Knox SA, Harrison CM, Britt HC, Henderson JV (2008). Estimating prevalence of common chronic morbidities in Australia. Med J Aust.

[27] Islam MM, Valderas JM, Yen L, Dawda P, Jowsey T, McRae IS (2014). Multimorbidity and comorbidity of chronic diseases among the senior Australians: prevalence and patterns. PLoS One.

[28] Department of Health and Ageing (2008). Impact of the Collection and Recording of PBS Under Co-payment Prescription Data: Final Report. Healthcare Management Advisors.

[29] *Fees, Patient Contributions and Safety Net Thresholds History of PBS Copayments and Safety Net Thresholds*. Available at: http://www.pbs.gov.au/info/healthpro/explanatory-notes/front/fee; Accessed on 25 August 2014

[30] *Home Care Service*. Available at: http://www.adhc.nsw.gov.au/individuals/help_at_home/home_care_service; Accessed on 28 August 2014

[31] Valderas JM, Starfield B, Sibbald B, Salisbury C, Roland M (2009). Defining comorbidity: implications for understanding health and health services. Ann Fam Med.

[32] Seah JZ, Harris A, Lorgelly PK (2013). Hospital Resource Use in Chronic Disease Combinations: Is It Enough to Just Add Them Up?. Value in Health.

[33] MacMahon B (1972). Concepts of multiple factors.

[34] Ware J, Kosinski M, Keller SD (1996). A 12-Item Short-Form Health Survey: construction of scales and preliminary tests of reliability and validity. Med Care.

[35] Australian Bureau of Statistics (2007). Household income and income distribution, Australia, 2005–06, Cat. no. 6523.0.

[36] Cunningham PJ (2009). Chronic burdens: the persistently high out-of-pocket health care expenses faced by many americans with chronic conditions. Commonwealth Fund pub.

[37] Belotti F, Deb P, Manning WG, Norton EC (2012). tpm: Estimating two-part models. The Stata Journal.

[38] Tisk JWR (2013). Applied longitudinal data analysis for Epidemiology.

[39] Cancer Voices Australia (2014). Senate Inquiry into Out-of-pocket costs in Australian Healthcare.

[40] Schoen C, Osborn R, Squires D, Doty MM, Pierson R, Applebaum S (2010). How health insurance design affects access to care and costs, by income, in eleven countries. Health affairs.

[41] Australian Institute of Health and Welfare (2011). Health expenditure Australia 2009–10.

[42] Xu K, Evans DB, Kawabata K, Zeramdini R, Klavus J, Murray CJ (2003). Household catastrophic health expenditure: a multicountry analysis. Lancet.

[43] Modugu HR, Kumar M, Kumar A, Millett C (2012). State and socio-demographic group variation in out-of-pocket expenditure, borrowings and Janani Suraksha Yojana (JSY) programme use for birth deliveries in India. BMC Public Health.

[44] Australian Bureau of Statistics (ABS) (2011). Health services: use and patient experience, cat. no. 4102.0, ABS, accessed 24 June 2014.

[CR45] The pre-publication history for this paper can be accessed here:http://www.biomedcentral.com/1471-2458/14/1008/prepub

